# Extending psychoradiology of emotion regulation from mental health to adaptiveness promotion

**DOI:** 10.1093/psyrad/kkaf037

**Published:** 2025-12-08

**Authors:** Tingting Wu, Jiajin Yuan

**Affiliations:** College of Physical Education, Xihua University, Chengdu 610039 Sichuan, China; The Laboratory for Affect Cognition and Regulation (ACRLAB), Institute of Brain and Psychological Sciences, Sichuan Normal University, Chengdu 610066, China

**Keywords:** emotion regulation adaptiveness, mental health, adaptive behavior, sport psychology, motor performance

## Abstract

Emotion regulation (ER) research has, for decades, focused on mental health outcomes, such as emotional recovery and wellbeing reinstatement, in behavioral, physiological, and neural measures across different regulatory strategies. Although important, the practical significance of ER research should not only be limited to mental health, but also needs to consider aiding people’s real-time adaptive behavior, to meet varying environmental demands or goals flexibly. In this paper, we propose an idea of ER adaptiveness that pays equal attention to both mental health outcomes, and how an ER strategy may be used to facilitate functional adaptiveness in meeting distinct goals. For instance, research of ER adaptiveness needs to highlight how to design regulatory strategies for the purpose of promoting cognitive, behavior, or social functions (nonaffective goal) in addition to that of affective wellbeing, and how to help a learned strategy work flexibly in changing contexts (affective goal). Lastly, taking application in sport psychology for example, we propose potential directions of how ER adaptiveness research may help participants to improve motor performance in competitive sports.

Starting from the initial proposal of a process model of emotion regulation (ER) (Gross, 1998; Gross and John, 2003; Gross and Thompson, 2007), there has been research interest from psychologists, cognigtive neuroscientists, and the broader scientific community on the consequences and mechanisms of ER in the last few decades. The psychoradiology of ER, such as using electroencephalography, physiological, or neuroimaging approaches to quantify the emotional consequences of regulatory strategy use, for example the use of distraction, cognitive reappraisal, or expressive suppression, have been intensely studied. In particular, the comparison of affective outcomes across different strategies has consistently remained in the spotlight of the field over the long term, receiving widespread attention. In early functional neuroimaging and physiological studies, researchers compared the use of instructed cognitive reappraisal with that of expressive suppression in multiple scales, including experiential emotion, peripheral physiological recordings, behavior of facial expression, prefrontal, insula, and amygdala activations as well as the time course of these neural structures, noting an advantage of cognitive reappraisal in decreasing emotional feeling, emotion-related sympathetic activation, and neural activations within affective neural circuits (Gross, 1998; Goldin *et al*., 2008; Buhle *et al*., 2014). Later, behavioral and eletrophysiological studies, using late positive potential as a key indicator, compared multiple strategies in the efficacy of unpleasant emotion regulation (Gallo *et al*., 2009; Paul *et al*., 2013; Yuan *et al*., 2015; Zhu *et al*., 2024). The results show a unique effect of expressive suppression in decreasing unpleasant emotion intensity or protecting mental health outcomes (e.g. depressive mood and hostibility) in German (Paul *et al*., 2013), Hong Kong and Mainland Chinese individuals (Soto *et al*., 2011; Yuan *et al*., 2015), but not in European Americans (Butler *et al*., 2007; Soto *et al*., 2011), while the use of distraction resulted in favorable regulatory effects across Chinese and European cultures (Paul *et al*., 2013; Li and Yuan, 2018). More recently, researchers of interpersonal ER have compared the efficacy of ER in improving affective outcomes between cognitive reappraisal and expressive suppression, in multiple measures from neural network analysis based on functional magnetic resonance imaging (fMRI) data to heart-rate synchrony and experiential measures (Liu *et al*., 2023; Wang and Shi, 2025).

On the other hand, in the past two decades researchers have also focused on automatic/implicit ER, either from the perspective of habitual use of a specific strategy or automation of strategy use by means of priming (Braunstein *et al*., 2017). This line of research aims at achieving emotion-regulatory goals without much cognitive depletion, and thus has important implications for affective rehabilitation in populations with impaired cognitive control functions (e.g. depressive patients). For the former, the links between the habitual use of a strategy (e.g. cognitive reappraisal and expressive suppression) and mental health outcomes (e.g. life satisfaction, self-esteem, or depressive symptoms) were regularly established based on questionaire reports (Gross and John, 2003; Soto *et al*., 2011; Gao *et al*., 2018). Then, regression models across these measures were established to determine the consequences of spontaneous use of a strategy on mental health outcome in the absence of effortful cognitive resource involvement, after controlling for other potential confounders. For instance, Gross and John (2003) illustrated that habitual use of reappraisal is linked to improved self-esteem, greater life satisfaction, and decreased depresive symptoms. For the latter, the goal of strategy use is often repeatedly primed prior to the exposure to emotionally evocative tasks/scenes, for the purpose of decreasing emotional reactivity without engagement of additional cognitive resource consumptions (Williams *et al*., 2009). Commonly used priming procedures include a sentence unscrambling task (Mauss *et al*., 2007; Williams *et al*., 2009), a synonym matching task (Wang and Li, 2017), explicit ER strategy training (Christou-Champi *et al*., 2015), or establishement of emotion-regulatory implementation intention (Gallo *et al*., 2009, 2012; Huang *et al*., 2020). A recent study showed that automatic reappraisal is in particular suitable for emotional reinstatement in patients diagnosed with major depressive disorder (Yuan *et al*., 2023), a population characterized by impaired executive control and frontal–parietal network functions (Joormann and Vanderlind, 2014; Li *et al*., 2024).

Despite the vital role of ER in mental health protection, ER studies should not be restricted to aspects of mental health outcome, which highlights emotional reinstatement and affective wellbeing (Harkness, and Hayden, 2018). Instead, in real-life settings, it is more important for ER research to serve the increase in adaptive functions, allowing individuals to meet varying enviornmental demands or personal goals flexibly across different contexts (Aldao *et al*., 2015). For example, in work settings, the use of suitable ER strategies to maintain emotional stability is, of course, important, which is a prerequisite to avoid emotion-triggered self-depletion and to focus limited resources on the stressful task at hand. However, what matters more, when we estimate the efficacy of a specific strategy, is whether the application of a given strategy can help us achieve the comprehensive optimal effects of emotional wellbeing and daily functions, such as increasing internal cognitive functions and external working performance. This is the key of the concept of ER adaptiveness, which highligts adaptive function/behavior promotion in addition to emotional recovery and wellbeing increases, as a consequence of ER strategy use. A potential real-life prototype of this argument is that the ER adaptiveness of a police officer at work should not only be manifested by his/her effective regulation of their own emotions, but more importantly should also be assessed by their achievement of investigation goals via working memory operations, attention involvement, affective thinking, etc. Fortuately, there are a few studies that have incorporated this idea during investigation of emotion-regulatory consequences.

For instance, Gao *et al*. (2024) investigated how cognitive flexibility training reduced depressive symptoms by proposing two potential pathways, one a context-covaried strategy selection (resembling ER flexibility in strategy choice, Aldao and Mennin, 2014) and the other an adaptiveness to meeting diverse goals, wherein the higher goal adaptiveness is represented by the balanced optimal effects of emotional improvement and cognitive performance promotion (working memory) during alternating goals. The findings of this study showed that the training of cognitive flexibility reduced depressive symptoms through enhancing goal adaptiveness (but not strategy choice flexibility) during ER. A plausible reason for this result is that cognitive flexibility, as a core element of executive function, subserves both the generation of cognitive reappraisal and the operations of working memory (McRae *et al*., 2012; Gu *et al*., 2025). This suggests that we need to reconsider the design of regulatory strategies in order to realize multiple adaptive goals apart from emotional downregulation itself, such as seeking a shared component subserving both reappraisal-based emotional reduction and memory promotion as described above (Gao *et al*., 2024). In line with this idea, we recently examined how cognitive reappraisal may be used to curb impulsive retweeting behavior of unconfirmed cyber-news in addition to downregulation of news-triggered emotions. As we aim to increase ER adaptiveness in terms of curbing impulsive retweeting in addition to emotional suppresion, the design of a reappraisal strategy not just highlighted reinterpreting the online news in a positive manner, but also highlighted “the cause of the event being unknown” simultaneously in order to reach dual goals (Xiao *et al*., 2024).

Aside from applying ER to pursing dual or multiple adaptive goals, the concept of ER adaptiveness also needs to consider endeavors to increase the applicability of a learned strategy to diverse situations outside of learning contexts. This is important to human adaptiveness as strategy learning is limited while emotional contexts vary all the time. Moreover, an adaptive ER processs (high in ER adaptiveness), as indicated by its literal meaning, also consists of the component of ER flexibility (Aldao and Mennin, 2014), namely flexible choice and application of appropriate strategies as a function of context alterations, because the use of a fixed strategy is evidently maladaptive with context changes (Sheppes *et al*., 2014). In line with this theme, several studies have investigated ER strategy choice across different contexts, such as how strategy preference varies flexibly depending on contexts of distinct valence/motivational intensities, depicting a likelihood of flexibile ER to achieve a balance of emotional outcome and cognitive resource involvement (Shafir *et al*., 2015; Yang *et al*., 2022). Also, there has been an attempt to examine how a learned regulatory strategy can be widely applied to different emotional contexts for the purpose of ER resource saving, such as the effects of increasing the coverage of the goal and the design of a flexible reponse, when emotion-related implementation intention is involved (Huang *et al*., 2020). In brief, it seems promising to shift the focus on mental health outcomes to seeking optimal effects of emotional wellbeing and adaptive behavior promotion.

However, existing research on ER adaptiveness remains scarce. As noted above, studies involving both emotional reinstatement and adaptive function promotion are restricted to a number of limited dimensions, such as memory function, strategy use flexibility, or cognitive resource saving. In fact, the ideas of ER adaptiveness should also be applied to many domains, such as motor impulsivity control of how ER may help decrease impulsive action (Zhao *et al*., 2023) as a result of emotion reduction, social interaction of how ER may promote one’s social initiative together with emotional reinstatement, and promotion of creative thinking.

For instance, sport psychologists are interested in how ER may help players keep emotional stability and, more importantly, promote motor learning, motor control, and flexibile motor decisions during a sports match. This is an important field where the concept of ER adaptiveness should be applied. A few studies have examined the effects of ER on motor performance. For instance, Bresin *et al*. (2012) reported that the upregulation of hostility impaired motor control accuracy at low, but not high, levels of agreeableness, suggesting that personality should be considered in the examination of ER and motor control association. Also, Beatty *et al*. (2014) compared three typical ER strategies during a ballistic pinch grip task, during which participants were required to produce a targeted pinch force at 10% of their maximal voluntary contraction. The results showed that attentional deployment resulted in the slowest reaction time, largest rate of force production, and poorest performance accuracy, while expressive suppression reduced the rate of force production and increased performance accuracy relative to emotional expression and attentional deployment. Closer to the theme of this paper, Beatty and Janelle (2020) proposed in a review that current ER studies put the main emphasis on mental health rather than sports performance, which hinders the application of ER to the coordination and execution of motor actions, as well as the enhancement of motor performance. It is worth noting that in sports involving whole body aerobic movement such as tennis or table tennis, motor performance should be quantified by objective indicators, such as gait initiation velocity, stepping velocity, stroke force, ball velocity, spin rate, and placement accuracy. As such, ER is highly necessary during a competitive match, wherein how to implement fast ER and how this regulation may support optimal performance at crucial points are both of interest and warrant quantitative investigation.

As noted above, suitable updating of a strategy design may help to realize dual goals as characterized by ER adaptiveness (Xiao *et al*., 2024). Therefore, to use the concept of ER adaptiveness for improvement of motor performance, we need to consider dual goals in the design of sports-related ER strategies. For example, we may highlight setting the ER goal of calmness, which counteracts competitive stress and anxiety, and the ways to reach it by linking potential stressors (e.g. setback) to an adaptive strategic response (e.g. positive self-talk and concentration) prior to the match. This method has proven effective in producing long-lasting automatic ER effects, without additional cognitive costs during actual stressor onset (Chen *et al*., 2021; Li *et al*., 2025). However, to maximally focus on the match itself, a player just needs to use positive self-talk and attend to the current moments during the match, without rethinking about the goal, whose online pursuit depletes limited resources and thus impairs self-regulation (Vohs *et al*., 2009). In this regard, future studies of ER adaptiveness in sport psychology should attach primary emphasis to the optimal effects of emotional wellbeing and motor/sports performance through designing regulatory strategies in accordance with contextual demands.
